# Acute Autonomic Symptoms with Anti-myelin-associated Glycoprotein Neuropathy as a Presentation of Small B Cell Lymphoma: A Case Report and Literature Review

**DOI:** 10.7759/cureus.3105

**Published:** 2018-08-06

**Authors:** Muhammad Umair Jahngir, Raghav Govindarajan

**Affiliations:** 1 Neurology, University of Missouri Healthcare, Columbia, USA; 2 Neurology, University of Missouri, Columbia, USA

**Keywords:** polyneuropathy, sensorimotor neuropathy, autonomic dysfunction

## Abstract

The association of symmetrical distal sensorimotor polyneuropathy with anti-myelin-associated glycoprotein antibodies (MAG) has been well established. Although autonomic symptoms are uncommon with anti-MAG antibody neuropathy (MAN). We are presenting an unusual case, who developed acute onset urinary retention, orthostatic hypotension, bradycardia and was found to have MAN. She was a 68-year-old lady, presented with progressive ascending numbness, weakness of her extremities and balance problems. On neurological examination, she had generalized muscle weakness, reduced perception to all modalities of sensation with marked impairment of vibration and position sense in the lower limbs. Reflexes were diminished throughout and Romberg sign was positive. Initial lab work including thyroid-stimulating hormone (TSH), vitamin B12, Hb1c, and routine lab reports was normal. The patient then developed acute urinary retention, severe orthostatic hypotension, and symptomatic bradycardia. Workup during this time revealed M spike on serum electrophoresis with IgM kappa on immunofixation. IgM titers were higher than normal. Initially, she was thought to have monoclonal gammopathy of undetermined significance (MGUS) related neuropathy but further workup showed very high levels of anti-MAG antibody titer. Further workup including a bone marrow biopsy revealed a small B cell lymphoma. Only a few cases have reported a small B cell lymphoma presenting with MAN-associated autonomic symptoms. She is currently being treated with rituximab with significant improvement in her neuropathic symptoms. Further case studies are needed to show whether autonomic symptoms are the feature of MAN or this atypical presentation is the paraneoplastic manifestation of the lymphoma.

## Introduction

Myelin-associated glycoprotein (MAG) is the most studied antigen in patients with neuropathy and IgM paraproteinemias [1]. Most of the patients with monoclonal gammopathy have IgM dysglobulinemia, and among them, about 2/3rd of the patients have antibodies against self-antigen like MAG [2]. Ten percent of these patients with idiopathic polyneuropathy in a period of one year have an underlying serum monoclonal gammopathy [2, 3].

Neuropathy can be the presenting complaint of the patients who are thought to have an autoimmune disorder caused by monoclonal immunoglobulin-like IgM M-protein. These antibodies, in the presence of complement proteins, have found to be the cause of axonal degeneration or demyelination by reacting with the proteins present in the central and peripheral nervous system [4]. These epitopes are biochemically glycoproteins, glycolipids, sulfoglucuronyl paragloboside (SGPG) or sulfoglucuronyl lactosaminyl paragloboside (SGLPG), and are present in both myelin and axons of peripheral nerves [5]. The antigenic component of myelin-associated glycoprotein lies in the carbohydrate part of the molecule, as deglycosylation of purified human myelin-associated glycoprotein causes it to lose its antigenicity [1].

Anti-MAG antibodies also co-react with other nervous system antigens, most importantly an antigenic glycolipid, which is identified as sulfoglucuronyl glycosphingolipid (SGPG). Unlike MAG, SGPG is only present in the peripheral nerves. Therefore, the more logical reasoning for peripheral symptoms in patients with anti-MAG peripheral neuropathy is the presence of glycolipid SGPG in the peripheral nervous system, to which all monoclonal anti-MAG IgM antibodies will react, and thus serve as a primary antigenic target. It is found that >50% of the IgM paraproteins recognize MAG and SGPG, and 2/3rd of them recognize the acidic glycolipids, making them the most common site for antigen-antibody cross-reactivity [1]. Anti-MAG antibodies have been found to cross-react with other antigens like sulfatide, and may come up as cryoglobulinemia which causes vasculitis on the clinical presentation [6].

The clinical presentation varies from purely sensory to purely motor or sensorimotor peripheral neuropathy [2, 5], with or without ataxia and tremors [1, 2, 7]. Motor involvement is usually late in the course of the disease. It is found to be a slowly progressive, and symmetrical distal neuropathy and is therefore named as distal acquired demyelinating symmetric neuropathy (DADS) [2]. Patients with anti-MAG neuropathy with motor deficits on clinical examination show mild to moderate weakness in extremities that typically appears first in toe extensors and takes several years to progress. The selective loss of myelination of larger nerve fibers is consistent with the clinical findings of impaired proprioception and sensory ataxia [1]. Autonomic symptoms are rarely seen, because of primary amyloidosis in the patients of paraproteinemias [8]. The tremors associated with anti-MAG neuropathy are difficult to treat. They may be disabling and respond to immunotherapy [1]. Few of the demographic facts are inferred by a retrospective study which showed that the mean age of diagnosis is 69 years, with the mean duration of symptoms before diagnosis is two years. It was found to be 2.7 times more common in males [2].

Demyelination pattern will be evident on nerve conduction studies which shows slowing of motor conduction, and decreasing sensory nerve action potentials (SNAPs), with no conduction block [2] and increased distal sensory and motor latencies [1, 7]. On nerve conduction studies, terminal latency index has been found to be reduced, because demyelination typically causes disproportionate prolongation of distal motor latencies and the proximal segment conduction velocities. The terminal latency index is calculated as: Distal distance/(motor conduction velocity x distal motor latency) [3]. The terminal latency index is found to be as low as 0.25, with no conduction block [9].

Signs of demyelination or axonal degeneration can be seen on nerve biopsies. Widening of lamellae of the myelin sheath is a pathognomic finding of anti-MAG polyneuropathy, as monoclonal IgM autoantibodies deposit throughout the myelin sheath, unlike other chronic inflammatory demyelinating polyneuropathies like Guillain Barre Syndrome. The pattern of demyelination is segmental and the widening of the lamella is found to be irregular through the course of nerve fiber [7]. Other factors that contribute in the human disease include antigenic modulation [10, 11], and inhibition of remyelination [12].

Other investigations need to be done when the diagnosis is equivocal on previous investigations or to provide the source of an additional evidence to support the diagnosis. The cerebrospinal fluid shows albumin-cytological disproportion in these patients, with protein levels of about >250 mg/dL [1]. Serum immunofixation electrophoresis is a preferred screening test as it has the higher sensitivity than that of serum protein electrophoresis to detect the M-proteins in the blood [13].

According to the Peripheral Nerve Society guidelines on the management of paraproteinemic demyelinating neuropathies, every patient with IgM paraproteinemia should have anti-MAG antibodies checked. If they turn out to be negative, other IgM antibodies against neural antigens including gangliosides GQ1b, GM1, GD1a, GD1b, and SGPG should be considered [9].

## Case presentation

A 68-year-old female presented with gradual onset of negative sensory symptoms like numbness, and weakness particularly on extremities bilaterally. She also had some balance problems for the same time duration. On neurological examination, there was diffusely reduced muscle strength of 4/5 on Medical Research Council (MRC) muscle power grading scale, along with the reduced perception to all modalities conducting either by dorsal column lemniscus or spinothalamic pathway. She also had some loss of sense of vibration and sense of proprioception peripherally. Moreover, there was a generalized hyporeflexia and gait examination showed a positive Romberg sign. On further inquiry, there was no previous history of similar symptoms or recent history of having any upper respiratory tract infection or diarrhea. There was no history of recent travel. Her current medications included losartan (50 mg) for her blood pressure control and the multi-vitamins. Her blood pressure was under control and lab results from the medical record of last year were normal. Initial workup for her unexplained neuropathy included serum TSH, vitamin B12, HbA1c along with routine baseline laboratory investigations, to rule out the more prevalent causes of these symptoms. These laboratory tests turned out to be normal. The patient then developed acute urinary incontinence and severe orthostatic hypotension. She also developed symptomatic bradycardia, severe enough to place a temporary pacemaker to relieve her symptoms.

Meanwhile, further workup was ordered which showed M spike on serum electrophoresis with IgM kappa on immunofixation. IgM titers were surprisingly high; 568 mg/dl (normal 40–230 mg/dl). Initially, the probable diagnosis was monoclonal gammopathy of undetermined significance (MGUS) related neuropathy. Hematological workup was then extending, which revealed anti-MAG antibody titers >1:102400 (normal < 1:1600). Bone marrow biopsy showed small atypical lymphoid cells which stained positive for CD20, PAX-5, with rare CD138 positive plasma cells. These findings were consistent with a small B-cell lymphoproliferative disorder. She is currently being treated with rituximab with significant improvement in her neuropathic symptoms. Acute autonomic symptoms can be a rare [8] and a confusing clinical manifestation of anti-MAG neuropathy.

## Discussion

We are presenting a rare presentation of MAG neuropathy. Autonomic features are rarely evident in these patients. Our patient first developed sensorimotor deficits symmetrically and later in the course of disease developed clinically significant autonomic symptoms.

Paraproteinemias are the bunch of disorders in which monoclonal plasma cells cause the proliferation and deposition of monoclonal M-proteins. The most common M-protein is IgG [3], but the most common paraprotein associated with neuropathies is IgM (approximately 50% of paraproteinemic neuropathies) [14]. MGUS is the most common paraproteinemia. MGUS (IgM-MGUS) and Waldenstrom Macroglobulinemia (WM) are the two paraproteinemias in which M-proteins are mainly IgM. About 33% of the patients with MGUS have associated peripheral neuropathy, and 50% of these patients have anti-MAG antibodies [3], more commonly associated with kappa than lambda light chains [9], and cross-react with the glycolipids and sulfatides like SGPG and sulfated glucuronyl paragloboside (SGLPG) on peripheral nerves [3]. The risk of conversion of MGUS into serious B-cell disorder in a year is estimated to be 1–2% [15]. The risk of conversion of MGUS into malignant disorders (like multiple myeloma, chronic lymphocytic lymphoma, IgM lymphoma, macroglobulinemia, plasmacytoma) is 12% at 10 years and 30% at 25 years [16]. WM is a B-cell proliferative disorder in which lymphoplasmacytic cells infiltrate the bone marrow. It will also cause distal symmetrical neuropathy, which is the presenting complaint in about 20% of the cases and is more often sensory [3]. Like MGUS, 50% of patients with WM-associated neuropathy have anti-MAG antibodies [8]. Anti-MAG neuropathy has also been reported with malignant lymphoproliferative diseases like B-cell lymphoma or chronic lymphocytic leukemia [9] and same is the case with our patient, whose final diagnosis was turned out to be B-cell lymphoma.

The associated risk of having autonomic symptoms like emesis, diarrhea, orthostasis, anhidrosis, xerosis, xerostomia, abdominal cramps, non-reactive pupils, and syncope [17], in patients with diagnosed MGUS was reported to be increased (RR 3.2, 95% CI: 1.3–8.3). The setback of this literature is that it did not look up for the type of M-proteins and the risk of having autonomic symptoms was partly because of the increased risk of primary (AL) amyloidosis in this population [8]. Twenty percent of the patients with AL amyloidosis have neuropathic symptoms and autonomic symptoms are more frequently seen in these patients [18].

A retrospective study was conducted. Patients participated in the study were presented with sensorimotor neuropathy (53%), with significant sensory ataxia (32%) and prominent sensory symptoms without ataxia (28%). The patients were then divided into two subgroups. The first group consisted (68.2%) of patients with typical clinical (sensory or sensorimotor symptoms), physiological (pattern of demyelination in the distal segments of nerves), immunological (higher titers of anti-MAG antibodies, >8000 BTU), and pathological (positive on immunofluorescence studies and widening of lamellae) profiles for anti-MAG neuropathy. While the second group included (31.6% patients) those with chronic inflammatory distal polyneuropathy like features that were demyelinating features on intermediate and proximal segments of nerves, preserved sensory potentials, low anti-MAG antibody titers, and absence of typical immunohistological findings. Intravenous immunoglobulin (IVIg) was tried but had caused only modest transient improvement in clinical symptoms. It was reported that IVIg gave more satisfactory results in patients with the motor phenotype of anti-MAG neuropathy [2].

Another cohort study was conducted to understand the pathophysiology and response to the treatment in the patients with anti-MAG neuropathy. It was found that patients with anti-MAG antibodies are heterogenous phenotypically and those with coexisted anti-MAG and anti-sulfatide antibodies, are clinically similar to anti-MAG neuropathy. The pathological and neurophysiological findings could differ in the patients with anti-sulfatide antibodies which leads to the fact of different response of these patients to the therapy. Thus, it is reasonable to screen the patients with anti-MAG antibodies for other possible paraproteinemias [7].

On immunohistochemistry, patients with neuropathy and anti-MAG M-proteins have shown immunoreactive deposits of IgM along the outer margins of the myelin sheath of peripheral nerves. It was interesting to find out the absence of complement components at the site of immunoglobulin deposits [4]. The same is the case with other antibody-mediated disorders like myasthenia gravis [19]. In order to prove the same context, a study was conducted after injecting the antibody containing subperineurial injection of patient’s serum containing anti-MAG antibodies into the endoneurial compartment of the cat's nerve, to assess the degree of demyelination after the injection. To induce the reaction, the human-derived anti-serum with added guinea pig complement was injected into the endoneurial compartment of the nerve. The transverse sections of the cat's sciatic nerve were then biopsied just proximal to the site of injection, at the second, fifth and eighth day of the injection containing anti-MAG IgM M-protein. On the second day, more than three-fourths of the myelinated nerve fibers showed splitting and graying of myelin sheath with a few mononuclear cells infiltration. While on the fifth day, biopsy showed that many myelinated nerve fibers were surrounded by Schwann cells, with myelin debris in their cytoplasm. Extensive vesiculation of the myelin sheath was seen and more extensive in the outer layers of the sheath. Macrophages were also visible with abundant cytoplasmic granules. On the eighth day of injection, most of the large axons were completely demyelinated [4]. Macrophages-associated demyelination is also seen in other chronic inflammatory distal polyneuropathy [2].

The concentration of M-protein was also found to be directly proportional to the degree and extent of demyelination. The role of the complement component was supported by the immunohistochemistry of the biopsy samples. C3 component of the classical pathway was found to be the contributory factor of demyelination within the injected nerve fascicles. The sural nerve biopsy in the patients with peripheral neuropathy and positive anti-MAG antibodies on Western Blot and/or enzyme-linked immunosorbent assay (ELISA), have shown deposits of IgM and the C3d component of the complement pathway. Two very interesting facts related to immunohistochemistry were, the extent and the quantity of complement component was more transient as compared to IgM M-proteins which persisted much longer in the course of nerve demyelination, thus complement activation was necessary to trigger the antibody-antigen reaction but did not require for the continued breakdown of the myelin. The vesicular changes in the inner layers of myelin sheath were typically evident along with the degenerative changes in the outer layer, as compared to the other myelin disorders in which only outer layers of myelin sheath would be affected. This phenomenon showed the injury of the myelin-forming cells rather than the direct insult to the myelin sheath. In actual human disease these M-proteins have found to enter the endoneurium continuously but in a concentration lower than that in serum, which causes gradual but persistent damage to the myelin sheath, thus results into chronic and progressive neuropathy [4]. The widening of lamellae of the myelin sheath is not found very often on biopsy but is specific for MAG neuropathy [5].

Another prospective study was conducted at the laboratory of a medical center on patients who presented with peripheral neuropathy and have positive anti-MAG or anti SGPG antibodies on western blot or ELISA testing. It was noted that 85.7% of patients were presented with purely sensory or sensorimotor manifestations and had highly elevated titers of anti-MAG and anti-SGPG antibodies. Their sural nerve biopsies more often depicted demyelination rather than axonal degeneration. As compared to those 14.3% patients with purely motor deficit had high titers of both anti-MAG and anti-SGPG antibodies, but antibodies were highly positive with MAG by Western blot rather than by ELISA. The nerve biopsies in these patients were more consistent with axonal degeneration. It was later inferred that patients with the highest titer of anti-MAG antibodies by ELISA had more evident demyelination as compared to axonal degeneration. As SGPG is found to be present on both axon and myelin, it was logical to find both axonal degeneration and demyelination in patients with positive anti-SGPG antibodies. One of these patients with predominantly motor deficits was diagnosed case of Amyotrophic Lateral Sclerosis (ALS), and had a positive titer for anti-SGPG antibodies only [5]. The increased incidence of monoclonal gammopathies in the patients of ALS has also been reported [20].

## Conclusions

Acute autonomic symptoms can be a rare and a confusing clinical manifestation of anti-MAG distal symmetric polyneuropathy and workup should include bone marrow biopsy. Thorough workup including bone marrow biopsy is needed to rule out underlying B cell lymphoma in the presence of a monoclonal gammopathy. Further case studies are needed to evaluate whether the autonomic symptoms are the result of concomitant amyloidosis in the patients with paraproteinemias and lymphoproliferative disorders or the consequence of paraneoplastic manifestations of these hematological malignancies.
